# Female-biased expression of long non-coding RNAs in domains that escape X-inactivation in mouse

**DOI:** 10.1186/1471-2164-11-614

**Published:** 2010-11-03

**Authors:** Björn Reinius, Chengxi Shi, Liu Hengshuo, Kuljeet Singh Sandhu, Katarzyna J Radomska, Glenn D Rosen, Lu Lu, Klas Kullander, Robert W Williams, Elena Jazin

**Affiliations:** 1 Department of Evolution and Development, EBC, Uppsala University, Uppsala, Sweden; 2 Department of Microbiology, Tumor and Cell Biology, Karolinska Institute, Stockholm, Sweden; 3 Department of Neurology, Beth Israel Deaconess Medical Center, Boston, MA, USA; 4 Department of Anatomy and Neurobiology, University of Tennessee, TN, USA; 5 Department of Neuroscience, BMC, Uppsala University, Uppsala, Sweden

## Abstract

**Background:**

Sexual dimorphism in brain gene expression has been recognized in several animal species. However, the relevant regulatory mechanisms remain poorly understood. To investigate whether sex-biased gene expression in mammalian brain is globally regulated or locally regulated in diverse brain structures, and to study the genomic organisation of brain-expressed sex-biased genes, we performed a large scale gene expression analysis of distinct brain regions in adult male and female mice.

**Results:**

This study revealed spatial specificity in sex-biased transcription in the mouse brain, and identified 173 sex-biased genes in the striatum; 19 in the neocortex; 12 in the hippocampus and 31 in the eye. Genes located on sex chromosomes were consistently over-represented in all brain regions. Analysis on a subset of genes with sex-bias in more than one tissue revealed Y-encoded male-biased transcripts and X-encoded female-biased transcripts known to escape X-inactivation. In addition, we identified novel coding and non-coding X-linked genes with female-biased expression in multiple tissues. Interestingly, the chromosomal positions of all of the female-biased non-coding genes are in close proximity to protein-coding genes that escape X-inactivation. This defines X-chromosome domains each of which contains a coding and a non-coding female-biased gene. Lack of repressive chromatin marks in non-coding transcribed loci supports the possibility that they escape X-inactivation. Moreover, RNA-DNA combined FISH experiments confirmed the biallelic expression of one such novel domain.

**Conclusion:**

This study demonstrated that the amount of genes with sex-biased expression varies between individual brain regions in mouse. The sex-biased genes identified are localized on many chromosomes. At the same time, sexually dimorphic gene expression that is common to several parts of the brain is mostly restricted to the sex chromosomes. Moreover, the study uncovered multiple female-biased non-coding genes that are non-randomly co-localized on the X-chromosome with protein-coding genes that escape X-inactivation. This raises the possibility that expression of long non-coding RNAs may play a role in modulating gene expression in domains that escape X-inactivation in mouse.

## Background

Emerging evidence indicates that the regulatory pathways underlying sexual differentiation result in phylogenetically widespread transcriptional sex-bias in the brain of organisms ranging from *D. melanogaster *to humans [1]. We earlier uncovered an evolutionary conserved signature of sex-biased gene expression in the cortex of catarrhine primates including humans [2]. These results suggested that sexual differences present in adult human brain are in part genetically controlled and not solely attributed to environmental differences between the sexes. Furthermore, we demonstrated that several genes on the Y-chromosome are expressed in many regions of prenatal human male brain [3], raising the possibility that expression of Y-linked genes may be partially responsible for sexual dimorphism during early development in the human brain. Moreover, sex-biased expression is partly conserved between mouse and human, suggesting that there is a common mechanism of transcriptional modulation of sex-bias operating across mammals.

The mechanisms by which sex differences in the brain are established and controlled during development are not understood in detail, but sex hormones clearly play major roles. However, sex hormones are not sufficient to explain all sex differences, and genes encoded in the sex chromosomes are also known to be important [4]. These genes include male-specific Y-encoded genes as well as female-biased X-encoded genes that escape the dosage compensating inactivation of one X-chromosome in females. In mouse, only a few genes that escape X-inactivation have been identified [5-9]. These genes are known to produce female-biased expression very early during development [10], but the molecular control mechanisms remain mostly unknown.

A genome-wide investigation of whole adult mouse brain and several peripheral tissues demonstrated that sexual dimorphism in gene expression is highly tissue specific [11]. However, sex-biased expression in distinct regions of the brain has not been previously investigated. Since the brain is a highly heterogeneous and functionally compartmentalized structure, it is of interest to explore whether gene expression in distinct regions of the brain is regulated differentially in males and females. Indeed, three-dimensional magnetic resonance microscopy in mice demonstrated sex-specific, post-puberty changes in brain structures [12]. Furthermore, sex-specific functional differences in various brain regions, particularly the amygdala and hypothalamus, have been observed in both human and rodent [13-15]. Given the implications of sex-biases for many neurological and psychiatric disorders [16], the importance of studying sexually dimorphic gene expression in the brain is increasingly being realized.

In the present study, we investigated male and female gene expression in distinct structures of the mouse brain using genome-wide microarray analysis. The study unravelled sub-structure-specific sexual gene expression dimorphism in the mouse brain. Our findings also include novel observations regarding the regulation of domains and genes escaping X-inactivation on the mouse X-chromosome.

## Results

### Sexually dimorphic gene expression in distinct brain regions

To investigate possible sub-structure-specific as well as potentially uniform sex-biased gene expression in the adult mouse brain, we assessed genome-wide RNA expression in striatum, neocortex, hippocampus and eye from male and female mice using oligonucleotide microarrays. Lung was included in the analysis to allow comparisons with a peripheral tissue. We reasoned that sex-biased transcription present both in brain and in a peripheral tissue would indicate wide-spread expression bias. This in turn would suggest a sex-biased control mechanism for these genes operating throughout the body and not specifically in the brain. Two microarray platforms were used: Illumina 6v1.1 in case of striatum and neocortex and Affymetrix M430v2.0 in case of hippocampus, eye and lung. A total number of 456 microarrays were included in the study, comprising a balanced number of male and female samples in each tissue (Table 1, Methods).

**Table 1:** Identification of sex-biased genes in five tissues

	**# Arrays**		**# Genes**			**# Genes on Sex Chr**					
**Tissue**	**Females**	**Males**	**Total**	**Female up**	**Male up**	**X Obs**	**X Exp**	**p**	**Y Obs**	**Y Exp**	**p**
			
**A**. Striatum	43	43	173	35	138	15	6.3	*	4	0.29	*
Neocortex	43	43	19	9	10	7	0.66	**	8	0.03	***
			
**B**. Hippocampus	60	60	12	8	4	8	0.4	***	4	0.008	***
Eye	60	60	31	21	10	12	1	***	5	0.021	***
Lung	22	22	160	101	59	16	5.4	**	5	0.11	***
											
Total	228	228									

**A: **Tissues analysed using Illumina 6v1.1 platform.

**B: **Tissues analysed using Affymetrix M430v2.0 platform.

**# Arrays: **Number of arrays used for each tissue and sex.

**# Genes: **Number of significant sex-biased genes in each tissue and sex. Selection criteria: Wilcoxon Mann-Whitney test followed by Benjamini-Hochberg (B-H) correction for multiple testing. B-H adjusted threshold p ≤ 0.05. Unannotaded probes and gene-probe redundancies are not included in these numbers; Additional file 1 includes all significant probes and raw p-values.

**# Genes on Sex Chr: **Observed number of genes on the sex chromosomes and expected numbers when "# Genes Total" are drawn from the array. Asterisks denote that X- and Y-linkage is statistically overrepresented among sex-biased genes according to Fisher's exact test. p-values: * ≤ 1E-3, ** ≤ 1E-10, *** ≤ 1E-15.

**Figure 1:**
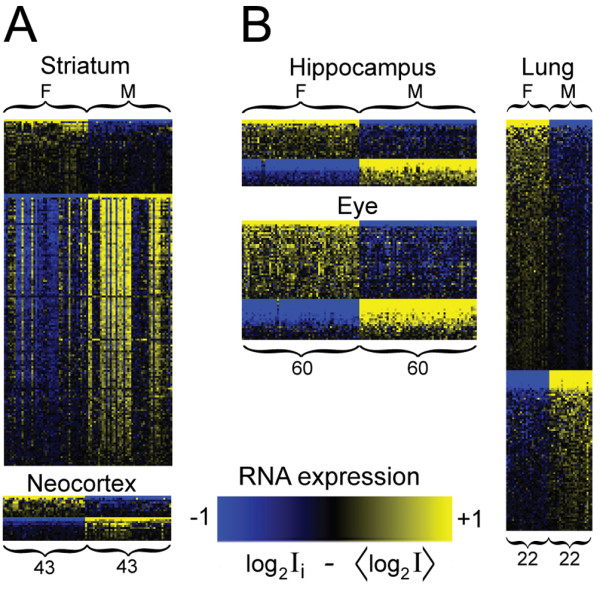
**Overview of significant probes in microarray analysis**. An overview is shown of the probes presenting significant sex-biased signals in five tissues. **A: **Tissues analysed using Illumina 6v1.1 platform (striatum and neocortex). **B: **Tissues analysed using Affymetrix M430v2.0 platform (hippocampus, eye and lung). Columns designate samples (F: females, M: males) and rows designate probes. Yellow colour denotes up-regulation and blue colour denotes down-regulation of a gene in a sample, as compared to the overall mean expression of the particular gene within a tissue in the sex-balanced data sets.

Genes with sexually dimorphic RNA expression were identified in striatum (n = 173), neocortex (n = 19), hippocampus (n = 12), eye (n = 31) and lung (n = 160) using FDR ≤ 0.05 as significance criteria (Table 1). The female to male fold changes in expression were statistically significant but small (between 1.1 and 2-fold) in terms of mean and median values for most genes (Figure 1, Additional file 1). Only *Xist *(Inactive X-specific transcript, female up-regulated), *Prl *(Prolactin, female up-regulated) and genes located on the Y-chromosome (male up-regulated) showed female to male changes greater than two-fold in any of the brain tissues analysed.

We also investigated the chromosomal localization of sex-biased genes and observed a significant overrepresentation of X- and Y-encoded genes in all tissues investigated (Table 1). Indeed, sexually dimorphic gene expression was almost exclusively restricted to genes located on the sex chromosomes in neocortex and hippocampus. Interestingly, striatum contrasted with the other brain regions in terms of the wide chromosomal distribution of sex-biased genes as well as in terms of quantity of biased genes, with an order of magnitude increase in the number of sex-biased genes (Table 1, Additional file 1). This result demonstrated that sex-biased gene expression is not uniform throughout the whole brain, but rather differentially pronounced in diverse brain structures. Since two similar but not identical microarray platforms were used in the study, direct comparisons of number of sex-biased genes and the estimated fold-changes of individual genes in each tissue should take differences between the platforms into account (See Discussion).

Gene ontology and disease association analysis demonstrated that sex-biased genes in striatum are mainly related to brain-specific functions (Additional file 2). Therefore, the results suggest a possible molecular background behind well-known functional sex-differences in striatum.

### Common signatures of sex-biased gene expression in different brain regions

**Figure 2:**
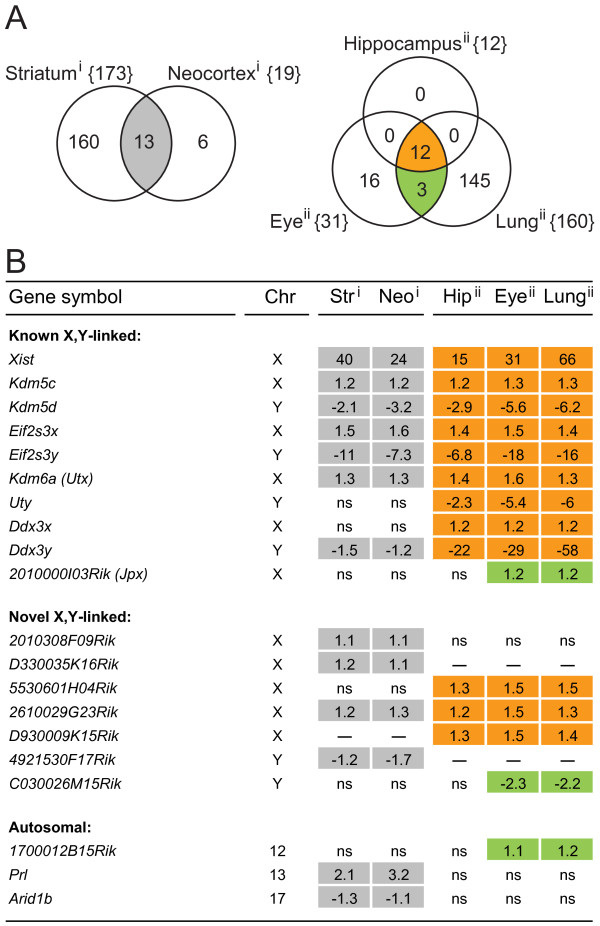
**Identification of genes with significant sex-biased expression in multiple tissues**. **A: **The Venn diagrams show the number of overlapping sex-biased genes in tissues within each array platform. **i: **Illumina 6v1.1 and **ii: **Affymetrix M430v2.0. The total number of sex-balanced genes in each tissue is given within brackets and the number of shared sex-biased genes in the tissues is given in the intersecting areas. All genes in the overlapping sets are named in panel **B**, shaded with the same colours as in the Venn diagrams. **B**: Genes that were sex-biased in more than one tissue in the microarray experiments are shown. Numbers denote mean fold changes, with positive signs denoting female up-regulation and negative signs denoting male up-regulation. The genes are divided into three categories: **Known X, Y-linked: **Genes on the sex chromosomes that were previously described as sex-biased, including *Xist*, genes that escape X-inactivation and their Y-linked paralogues. Also included is *2010000I03Rik *(*Jpx*), a gene located in the X-pairing region. **Novel X, Y-linked: **Genes on the sex chromosomes not previously described as sex-biased. **Autosomal: **Sex-biased genes located on the somatic chromosomes. Abbreviations: Chr: Chromosome, Str: Striatum, Neo: Neocortex, Hip: Hippocampus, ns: not significant, "--": not available on the array platform.

We investigated whether we could identify genes that are sex-biased in multiple tissues. The inventories of sex-biased genes in each tissue resulted in five lists (Additional file 1A-E). These lists were compared to identify genes that were sex-biased in more than one of the tissues included in the study (Figure 2A). We found that nearly all of the genes that were sex-biased in multiple tissues are located on the X- and Y-chromosomes. Many of these sex chromosome encoded genes, listed as "Known X, Y-linked" in Figure 2B, are already well documented in the literature in terms of sex-biased function and/or expression [9,17-22]. These genes include *Xist*, which is essential for the onset of X-inactivation [23], *2010000I03Rik *(also known as *Jpx*) which is located in the X-pairing region [24,25] and several genes previously known to escape X-inactivation, including *Kdm5c*, *Eif2s3x*, *Kdm6a *(formerly termed *Utx*) and *Ddx3x*. All X-linked genes named above were female-biased. We also detected male-biased expression of their Y-linked paralogous genes: *Kdm5d*, *Eif2s3y*, *Uty *and *Ddx3y*. In addition to previously reported sex-biased genes, we identified five novel X-linked genes with up-regulation in female tissues: *2010308F09Rik, D330035K16Rik*, *5530601H04Rik, 2610029G23Rik*, and *D930009K15Rik *("Novel X, Y-linked", Figure 2B). On the Y-chromosome, we identified two novel male-biased genes: *4921530F17Rik*, up-regulated in male neocortex and striatum and *C030026M15Rik*, up-regulated in male eye and lung tissues. Among the genes encoded in autosomal chromosomes, we did not identify any transcript that was sex-biased in all brain structures (Figure 2B). Two autosomal genes were however sex-biased in both striatum and neocortex: *Prl *(Prolactin, chr13, female up-regulated), a gene that promotes lactation, and *Arid1b *(AT rich interactive domain 1B, chr17, male up-regulated), a chromatin-remodelling factor. *1700012B15Rik *(unknown function, chr12, female up-regulated) was identified in both eye and lung.

Thus, we conclude that genes commonly sex-biased in multiple brain structures are primarily attributed to the sex chromosomes. Since most of the novel sex-biased genes that were differentially expressed in several parts of the brain are encoded in the X-chromosome, and since X-gene dose effects may indeed produce changes in brain function and behaviour [26], we restricted further analysis to female-biased X-linked genes.

### Novel female-biased X-encoded genes code for long non-coding RNAs

The function of the five newly identified X-linked female-biased genes, *2010308F09Rik*, *D330035K16Rik, 5530601H04Rik, 2610029G23Rik *and *D930009K15Rik*, is not known. Cross-species BLAST indicated that only *2610029G23Rik *is conserved between mouse and human (human gene symbol: Cxorf26). The lack of human homologs for the other four genes suggested that they could be non-coding. To explore this possibility, we performed coding potential calculations based on sequence composition [27]. This analysis classified *2010308F09Rik, D330035K16Rik *and *D930009K15Rik *as non-coding (Table 2A). Furthermore, *5530601H04Rik *and *D930009K15Rik *were found as annotated non-coding RNAs (ncRNAs) in two ncRNA databases, namely RNA-db [28] and NONCODE [29] (Table 2B). The classification of the female-biased transcripts as non-coding was further strengthened by the lack of any significant hit of their sequences in the mouse Refseq protein database (Table 2C). Thus, four out of five of the newly identified female-biased X-linked transcripts are most likely long non-coding RNAs (lncRNAs), a class that is generally defined as ncRNAs longer than 200 nucleotides [30,31].

**Table 2:** Coding/non-coding property of female-biased X-linked genes

		**A**. **CPC**		**B**. **nRNA db**		**C**. **BLAST x**	
**Gene symbol**	**Refseq RNA**	**Coding/Non-coding**	**Score**	**RNA-db**	**NONCODE**	**Cov, Ident**	**RefSeq Protein**
			
**Known X-linked:**							
*Xist*	NR_001463.3	weak coding	0.45	Y	Y	6, 28	XP_001478155.1
*Kdm5c*	NM_013668.3	coding	17.40	N	N	73, 100	NP_038696.2
*Eif2s3x*	NM_012010.3	coding	3.63	N	N	39, 100	NP_036140.1
*Kdm6a*	NM_009483.1	coding	16.02	N	N	80, 100	NP_033509.1
*Ddx3x*	NM_010028.3	coding	9.69	N	N	38, 100	NP_034158.1
*2010000I03Rik*	NR_015508.1	weak coding	0.14	Y	Y	11,68	XP_0014781580.1
							
**Novel X-linked:**							
*2010308F09Rik*	AK008545.1	noncoding	-1.13	N	N	20, 60	XP_001479963.1
*D330035K16Rik*	AK084720.1	noncoding	-1.21	N	N	18, 24	NP_035953.3
*5530601H04Rik*	AK164650.1	weak coding	0.83	Y	Y	2, 78	XP_001478711.1
*2610029G23Rik*	NM_026312.4	coding	3.05	N	N	22, 100	NP_080588.1
*D930009K15Rik*	AK148627.1	noncoding	-1.22	Y	Y	5, 29	NP_032530.1

**A: **Coding potential calculation (CPC) and coding/non-coding classification based on sequence composition [27].

**B: **Genes that are annotated as non-coding RNAs in ncRNA databases, namely RNA-db [28] and NONCODE [29]. (Y: yes, N: no).

**C: **Highest ranked hits when performing NCBI BLASTx using all 6 frames in Refseq protein database. Cov: % coverage of RNA transcript, Indet: % sequence identity.

### Female up-regulated chromosomal domains that contain a lncRNA and a protein-coding gene that escapes X-inactivation

**Figure 3:**
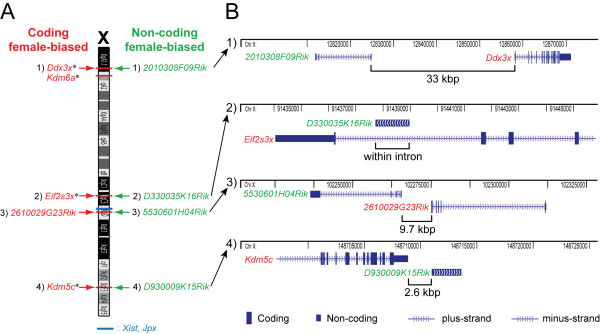
**Female-biased coding and non-coding genes are clustered on the X-chromosome**. **A: **Chromosomal locations (NCBI37/*mm9 *genome assembly) of the known and novel X-encoded female-biased genes presented in Figure 2B. Known coding X-inactivation escapees are marked with "*". **B: **Higher resolution map, showing that coding and non-coding female-biased genes are proximally paired on the X-chromosome.

All of the previously known female-biased genes on the X-chromosome that were identified in multiple tissues ("Known X, Y-linked", Figure 2B) escape, or are involved in the regulation of, X-inactivation [9,23-25]. Of particular interest was that when we investigated the chromosomal localizations of the novel female-biased lncRNAs, we observed that all of these non-coding genes are positioned close to female-biased coding genes on the X-chromosome (Figure 3A). Moreover, a closer examination revealed that three out of four female-biased lncRNAs are proximally paired with female-biased coding genes that are known to escape X-inactivation (Figure 3B). Indeed, *2010308F09Rik *is located 33 kbp upstream of *Ddx3x *and these two genes are encoded in a "head to head" orientation; *D330035K16Rik *is positioned within an intron of *Eif2s3x *and *D930009K15Rik *is located 2.6 kbp downstream of *Kdm5c*. Furthermore, the remaining female-biased non-coding gene, *5530601H04Rik*, is co-localized with the protein-coding and female-biased gene *2610029G23Rik*, and these genes are situated in a "head to head" arrangement separated by 9.7 kbp (Figure 3B).

**Figure 4:**
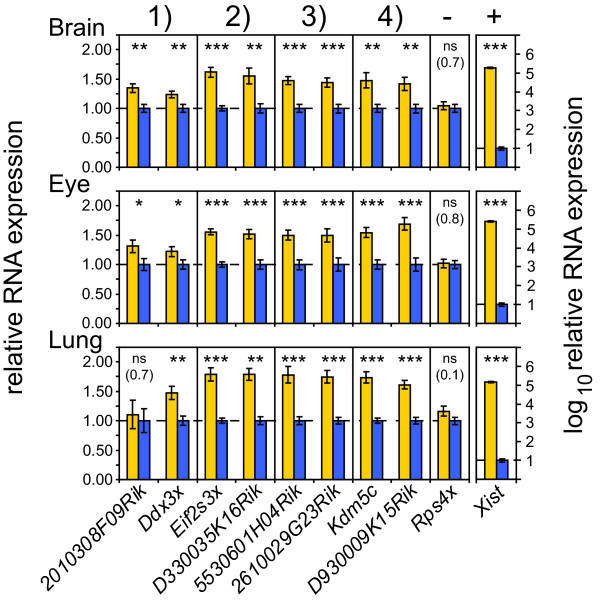
**Female-biased expression of pairs of coding and non-coding X-linked genes is validated by quantitative RT-PCR**. The RNA expression of co-localized pairs of coding and non-coding genes in three tissues, normalized to *Actb *and *Gapdh *is shown. **Brain **(n = 19 females, 19 males), **Eye **(n = 16 females, 16 males) and **Lung **(n= 16 females, 16 males). The X-inactivated gene *Rps4x *is used as a negative control, while *Xist *is taken as a female-specific control. The heights of the bars represent mean female expression (yellow bars) as relative to mean male expression (blue bars). Error bars signify +/- standard error of the mean. p-values: * ≤ 0.05, ** ≤ 0.01, *** ≤ 0.001 (two-sided unequal variance t-test), ns: not significant, non-significant p-values are given within brackets. The numbers 1-4 above the figure correspond to the four female-biased clusters in Figure 3.

Quantitative RT-PCR experiments confirmed the female-biased expression of the above mentioned pairs of X-linked coding and non-coding genes in brain, eye and lung tissues (p ≤ 0.05, two-sided unequal variance t-test) (Figure 4). One exception was *2010308F09Rik*, not confirmed as female-biased in lung, but evidently female-biased in brain and eye. Female to male fold-changes were in the same range as detected in the microarray experiments, and in the expected range for X-inactivation escapee genes [32]. An X-linked negative control for sex bias, *Rps4x*, which is inactivated on the silent mouse X-chromosome [33,34] and therefore not expected to show female-biased expression, remained unchanged. A positive control for female-bias, *Xist*, was highly female-biased (~10 000-fold) as expected.

The observation of female-biased domains in the mouse X-chromosome, containing a lncRNA and a protein-coding gene that escapes X-inactivation, suggested that the female-biased gene pairs in each domain might be co-regulated. Given the well known X-inactivation status of three out of four of the coding genes, we suspected that the three co-localized lncRNAs might also escape X-inactivation. We could not find any data in the literature concerning the possibility that the fourth female-bias cluster, containing the coding gene *2610029G23Rik *and the non-coding gene *5530601H04Rik *may also escape X-inactivation. Thus, we decided to study this cluster of two female-biased genes in more detail as described in the next section.

### A novel domain that escapes X-inactivation in mouse

**Figure 5:**
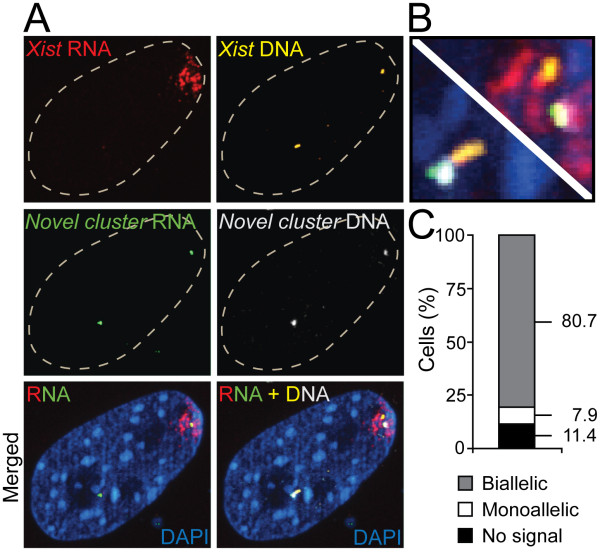
**A novel domain in the mouse X-chromosome that escapes X-inactivation**. Results from sequential RNA-DNA FISH experiments on the novel female-biased cluster containing *5530601H04Rik *and *2610029G23Rik*, and on *Xist*, in female mouse cells are shown. **A: **Confocal images, showing monoallelic *Xist *RNA signals (red) and biallelic RNA signals from the novel female-biased cluster (green). In agreement with the expected genomic distance between the *Xist *locus and the novel cluster (~1.6Mbp), DNA signals from *Xist *(yellow) appear close to the DNA signals from the novel cluster (white). RNA and DNA signals for the novel cluster are proximal, indicating that the intended nascent RNA was targeted. The nucleus is stained with DAPI (blue). **B: **Enlargements of the merged RNA + DNA image in panel **A**, showing that RNA and DNA signals for the novel cluster are overlapping. **C: **Quantitative data showing allelic expression of the novel escapee cluster in nuclei positive for *Xist *RNA (n = 88).

To investigate whether the chromosomal domain containing genes *2610029G23Rik *(coding) and *5530601H04Rik *(non-coding) escapes X-inactivation, we performed RNA FISH experiments in mouse cells, using a probe aimed at nascent RNA from the genomic cluster that contains the *5530601H04Rik *and *2610029G23Rik *transcripts. In parallel, we included a probe for *Xist *RNA. We counted signals in 88 randomly chosen cells in which *Xist *signals were detected. As expected, strong monoallelic *Xist *signals were distinguished (Figure 5A-B), marking the silenced X-chromosome. In contrast, signals from the novel female-biased cluster were biallelic in 80.7% of the cells, monoallelic in 7.9% of the cells and 11.4% of the cells lacked signals (Figure 5C). To verify that the detected nascent RNA signals were derived from the locus containing *5530601H04Rik *and *2610029G23Rik*, we subsequently performed DNA FISH with probes aimed to their genomic locus and a second probe aimed to the *Xist *locus (located at 1.6 Mbp distance from the two Riken genes). Indeed, RNA-DNA FISH experiments demonstrated that RNA and DNA signals for the female-biased cluster were overlapping (Figure 5A-B). Moreover, DNA signals for the female-biased cluster and the *Xist *locus appeared close, as expected given their short chromosomal distance. Altogether, these results demonstrated that the newly identified female-biased cluster contains genes that escape X-inactivation in the majority of the cells.

**Figure 6:**
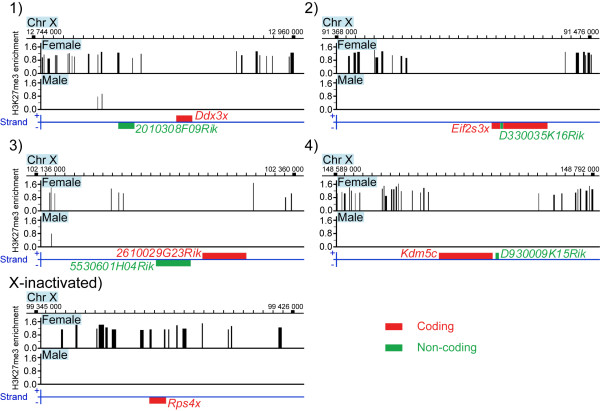
**Depletion of H3K27 tri-methylation in domains containing female-biased coding and non-coding genes**. The figure shows enrichment of the repressive histone mark H3K27me3 in female and male mouse liver in the identified domains containing a coding X-inactivation escapee gene and a female-biased lncRNA, and the neighbouring regions. *Rps4x *is included for comparison with an X-inactivated gene. Red colour designates coding genes and green colour designates non-coding genes. The numbers 1-4 above the graphs correspond to the four female-biased clusters in Figure 3 and 4. GEO accession: GSE20617, NCBI37/*mm9 *genome assembly.

Biallelic expression of this novel cluster strengthens the association between female-biased lncRNAs and domains that escape X-inactivation, and supports the possibility that female-biased lncRNAs are biallelically transcribed. Furthermore, this is supported by the lack of repressive chromatin mark, namely H3K27 tri-methylation (H3K27me3), in the respective loci (Figure 6). H3K27me3 is apparently depleted not only in protein-coding X-inactivation escapee genes, but also in the co-localized female-biased lncRNA genes. One of the lncRNAs, *2010308F09Rik*, was located in the border region of H3K27me3 enrichment. This might explain why female-bias of this transcript was not detected in lung tissues, and why the female to male expression ratio of the co-localized gene *Ddx3x *was consistently lower than the ratios for the other X-inactivation escapees in the quantitative RT-PCR analysis (Figure 4). We propose that female-biased lncRNAs transcribe from domains that escape X-inactivation in mouse, suggesting possible control functions of lncRNAs in such domains.

## Discussion

### Sex-biased gene expression differs regionally in the adult mouse brain

We observed a large difference in number and chromosomal localization of sex-biased genes between striatum and the other brain structures investigated, including neocortex, hippocampus and eye. These results suggest that sex differences are spatially regulated in distinct regions of the brain. A caveat to our tissue comparisons is that two similar but not identical array platforms were used in the study. Therefore, "microarray platform" cannot be excluded as a confounding factor when making direct comparisons of number of sex-biased genes over the platforms. Nevertheless, striatum clearly differentiated from neocortex in the amount and chromosomal locations of sex-biased genes, and these two tissues were analysed using the same platform. Our results thus indicate that sex-biased gene expression is brain region-specific rather that uniformly controlled in the whole brain. Whether only striatum, or also other additional regions are more sexually dimorphic than neocortex in terms of gene expression in the brain remains to be clarified. Indeed, the possibility that specific sex differences in brain gene expression may occur in a spatially regulated manner has earlier been proposed based on the high heterogeneity in structure and function of the brain [11]. We propose that further analysis of regions well-known to be functionally dimorphic in males and females, such as the hypothalamus [35] and the amygdala [36], may show more pronounced sex-biased gene expression.

The chromosomal distribution of sex-biased genes in striatum contrasted with the observations in neocortex and hippocampus. In these tissues, sex-bias was essentially restricted to genes encoded in the X- and Y-chromosomes. Striatum is perhaps best known for its involvement in regulation of movement, reward and emotions, and studies in rodents showed sex differences in functions that are dependent on this brain structure [37-39]. The results of our study provide molecular support for the notion that striatum may be essential in controlling at least some aspects of sexually dimorphic behaviours and functions.

A general conclusion from our study of discrete brain regions is it that most female to male gene expression differences are less than two-fold. These results are in agreement with measurements obtained in whole mouse brain [11] also showing expression differences less than two-fold for most genes. Although small in magnitude, these expression changes are statistically highly significant. A challenge for the future is to functionally analyse possible consequences of these sexual gene expression biases in different regions of the brain, their establishment during brain development and their regulation by hormones and hormone-independent factors.

### Sex-biased expression of sex-linked genes throughout the brain

Genes that were sex-biased in all brain regions included in the study were encoded exclusively in the X- and Y-chromosomes. This suggests that sex-biased transcription throughout the adult brain is restricted to sex-linked genes. The sex-specific functions of these genes in the brain should be the subject of future analysis.

Among autosomal genes, only two genes, namely *Prl *and *Arid1b*, were sex-biased in more than one brain region. Prolactin has more than 300 separate functions in vertebrates [40] and sex-biased functions are included among them. *Arid1b *is a chromatin-remodelling factor with widespread expression [41,42]. Increased expression of this gene in male brain, and its possible implications for sex-biased chromatin remodelling, remains to be investigated.

### Female-biased expression of lncRNAs in chromosomal domains that escape X-inactivation

One of the most important outcomes of our study is the identification of four female-biased lncRNA genes that are localized on the mouse X-chromosome close to female-biased protein-coding genes. These results define four female up-regulated chromosomal domains each of which contains a coding and a non-coding gene. A second important observation is that each of these X-chromosome domains contains genes that escape X-inactivation. Indeed, three out of four female-biased coding genes (*Ddx3x*, *Eif2s3x *and *Kdm5c*) were shown to escape X-inactivation by other investigators [9]. Our RNA FISH experiments demonstrated that also the fourth coding gene, *2610029G23Rik*, as well as the non-coding *5530601H04Rik*, are localized in a genomic region that escapes X inactivation. In addition, while the present article was under preparation, a second study demonstrated independently that *2610029G23Rik *escapes X-inactivation in a hybrid *Mus musculus × Mus spretus *cell line [43]. The non-random clustering of coding escapees and lncRNA genes on the X-chromosome (FDR = 0.00, Methods), the female biased expression of all of these genes, and the observed depletion of the repressive chromatin mark H3K27me3 in coding as well as in non-coding loci, supports the view that both coding and lncRNA genes escape X-inactivation. In conclusion, our study indicates the presence of four domains in the mouse X-chromosome, containing a protein-coding gene and a lncRNA, each of which escapes X inactivation.

Previous studies of X-inactivation in humans showed that escapee genes are grouped in large gene clusters on the X-chromosome [21,44-46], suggesting that escape mechanisms control continuous chromosomal domains. In mice, on the other hand, previously described escapee genes were interspersed and few, indicating that escape mechanisms control smaller regions or even single genes [9,21,46-48]. Our results suggest that clusters of genes that escape X-inactivation also exist in mice, although the size of these domains is apparently smaller than in humans. Escape mechanisms may be regulating both coding and non-coding genes collectively within confined domains in the mouse X-chromosome. It is possible that the female-biased lncRNAs escape X-inactivation as a side effect of being located closely to functional protein-coding escapee genes. However, an alternative possibility is that the lncRNAs themselves serve a function in the escaping domains, and this possibility will be discussed in the following section.

### Possible roles for long non-coding RNAs in domains that escape X-inactivation

The mechanisms that control escape from silencing on the inactive X-chromosome remain cryptic. Our observation of four chromosomal domains, each containing a female-biased coding and a non-coding gene, raises the possibility that lncRNA are themselves involved in the regulation of X-chromosome domains that escape inactivation. Although the functions of most lncRNAs remain enigmatic, there is increasing evidence suggesting roles in epigenetic regulation [30,31,49]. Recent reports showed that expression of lncRNAs can remodel local chromatin and promote transcriptional activation of closely located genes [50]. For example, the expression of *fbp1 *in yeast was shown to be mediated by expression of 5' located lncRNA transcripts, and accompanied by progressive opening of proximal chromatin [51]. Another example is the activation of yeast *pho5*, where the transcription of an intergenic, 3' located, antisense lncRNA contributed positively to chromatin plasticity and nucleosome disassembly [52]. Expression of lncRNAs in domains that escape X-inactivation might regulate the expression of neighbouring protein-coding genes by similar mechanisms, involving relaxation of nucleosome complexes, increased accessibility for transcriptional machineries and recruitment of transcription factors that activate transcription in the domain.

Other mechanisms of lncRNA control in X-inactivation escaping domains are possible. For example, lncRNAs may mediate movement of escapee domains to transcriptionally active compartments in the nucleus. Similar lncRNA-dependent movements were previously described, although towards transcriptionally silent compartments [53,54]. Another described role for lncRNAs involved the establishment of chromosomal boundaries between transcriptionally active and inactive domains [55,56]. By analogy, it is possible that transcription of lncRNA in escaping domains mediates the formation of chromatin boundaries to restrict the spread of heterochromatin from neighbouring silent regions in the X-chromosome, thereby ensuring escape of the co-localized protein-coding genes.

The proposed hypotheses could be tested in the future by mutation or silencing of the lncRNAs identified in our study.

X-linked lncRNAs are known to be involved in multiple steps of control of the binary transcriptional state of the X-chromosomes in mammalian females, including initiation, establishment and maintenance of X-chromosome silencing [57,58]. The current report raised the possibility that lncRNAs might act as gene expression modifiers in domains that escape X-inactivation. Unlike proteins and small RNAs, lncRNAs can remain tethered to the site of transcription, and therefore uniquely direct allelic regulation [57]. This property could favour the choice of lncRNAs as regulators of sexually antagonistic genes on the X-chromosome during evolution. Selection for cis-acting modifiers of expression of sexually antagonistic genes on the X-chromosome is certainly predicted [59]. lncRNAs as local expression modifiers in domains that escape X-inactivation may very well prove to be a novel example of such cis-acting elements in the X-chromosome.

Sex chromosomes have evolved from autosomes independently many times in animals [60]. However, the main dilemma of dosage compensation and regulation of sexually antagonistic genes has been alike for each sex chromosome system [61]. It is possible that lncRNAs may have evolved as a solution for allele-specific regulation of confined domains in several types of sex chromosomes. The fast sequence evolution of non-coding RNAs [62] could make these molecules suitable for regulation of sexually antagonistic genes. To evaluate this possibility, the roles of lncRNAs in allele-specific regulation of genes encoded in the sex chromosomes should be studied not only in mouse but also in species with other sex chromosome systems.

## Conclusions

Our data indicate that the degree of sex-biased gene expression varies between distinct brain structures in adult mouse brain. In particular, striatum showed wider transcriptional sex-bias than neocortex both in terms of number of sex-biased genes and chromosomal distribution of these genes. More interestingly, we identified female-biased lncRNA genes clustered on the X-chromosome with protein-coding genes that escape X-inactivation. These clusters are free of repressive histone marks, supporting the view that female-biased lncRNAs are biallelically expressed in domains that escape X-inactivation. Based on our observations, we propose that these lncRNAs might modulate the epigenetic state of nearby coding genes.

## Methods

### Identification of sex-biased genes

*Microarray data:* Microarray collections from discrete tissues were contributed by GeneNetwork (GN) (University of Tennessee). Details of the processing of tissues and the array data are described earlier [63-66], and summarised on the GN homepage http://www.genenetwork.org. In brief, Illumina Mouse-6v1.1 arrays (striatum and neocortex) were normalized with rank invariant normalization and Affymetrix M430v2 arrays (hippocampus and eye) were normalized with robust multichip average (RMA). Signals were log transformed and standardized to a mean of 8 and a standard deviation of 2. RMA normalization was also applied to the lung data (Affymetrix M430v2). Array data can be accessed via GN http://www.genenetwork.org. GN Striatum data set: "HQF BXD Striatum Illumina Mouse-6.1 November 2007 Rank Invariant Data Set", GN Accession: GN152. GN Neocortex data set: "HQF BXD Neocortex ILM6v1.1 (Feb08) RankInv", GN Accession: GN157. GN Hippocampus data set: "Hippocampus Consortium M430v2 (June06) RMA", GN Accession: GN110. GN Eye data set: "Hamilton Eye Institute Mouse Eye M430v2 Data Set (Sept08) RMA", GN Accession: GN207. GN Lung data set: "HZI Lung M430v2 (Apr08) RMA", GN Accesion: GN160. The analysis was restricted to include only C57BL/6 × DBA/2 mice. Prior to any further analysis, the data collection was screened for arrays in which sex had been potentially reversed in the databases, since such misclassifications would distort and reduce power of the sex-specific analysis. This was done by graphing the log_2_ intensity values of two *Xis*t probes in each array and tissue in scatter plots. Male and female arrays in which *Xist *expression fell in the expected quadrant of the opposite sex were excluded from the analysis (Additional file 3). The data sets were subsequently balanced to include an equal number of arrays from each sex. *Identification of sex-biased genes: *Sex-biased genes were identified in each tissue using the Wilcoxon Mann-Whitney test followed by Benjamini-Hochberg correction of the p-values (TIGR MeV v.4.3 package [67]). The nonparametric Wilcoxon test avoids possible violations of assumptions of normal distribution of signals in each particular probe-set, while the Benjamini-Hochberg correction hinders inflation of false positives in the resulting gene lists, which may otherwise be a caveat in microarray approaches. The criterion for differential expression was a Benjamini-Hochberg adjusted p-value lower or equal to 0.05. Both raw and adjusted p-values for each significant probe are listed in Additional file 1. Heatmaps were generated in TIGR MeV v.4.3 [67]. Gene ontology and disease association analysis, presented in Additional file 2, was performed using Ingenuity Pathway Analysis Software v8.6 (Ingenuity Systems). FDR of the observed co-localization of female-biased genes on the X-chromosome was calculated by considering *Xist, Jpx *and the coding escapee genes (coding genes in Figure 3) as fixed and repeatedly drawing four random X-linked probes from the arrays (since four lncRNAs were identified). FDR was calculated as the number of times four drawn probes were located at a maximum of 50kb distance up-/down-stream of the fixed genes divided by the number of permutations (10^4^).

### Quantitative RT-PCR

*Mouse tissues:* Tissues were collected from female and male C57BL/6 × DBA/2 mice, and these samples were independent from the individuals used in generating the microarray data. Animals had been housed at Uppsala Biomedical Center, Sweden, in agreement with animal research ethical regulations (Swedish ethical committee permit: c79/9). Dissected brain (rostral part, including striatum, neocortex and parts of hippocampus, excluding olfactory bulbs), eye and lung tissues were snap-frozen on dry ice and subsequently kept at -80°C. *RNA and cDNA*: RNA was extracted using Trizol (Invitrogen) according to the manufacturer's instructions. To inspect RNA quality, 28 S and 18 S ribosomal bands were examined by gel electrophoresis, and optical 260/280, 260/230 ratios were measured (ND-1000, NanoDrop Technologies) ranging 2.0-2.2. RNA was reversely transcribed to cDNA using a Dynamo cDNA synthesis kit F-470L (Finnzymes) and the following reagents: 0.95 μg total RNA, 15 ng/μl random hexamers, 10 U/μl M-MuLV RNase H- reverse transcriptase, 1 × RT buffer, nuclease-free water, in a total reaction volume of 20 μl. Incubations were performed in a PTC-100 Peltier Thermal Cycler (MJ Research): 25°C; 10 min, 37°C; 45 min, 85°C; 5 min. cDNA samples were subsequently diluted 1:10 in double distilled water and kept at -20°C until usage. *Primers: *Transcript-specific primers (Additional file 4) were designed on opposite sides of exon/exon junctions using Primer3 [68]. Since *D330035K16Rik *and *D930009K15Rik *lack exon/intron structures, intra-exonic primers were used for these transcripts. Multiple primer pairs were tested for each transcript, and primers with the highest PCR efficiencies were selected for the experiments. *qPCR: *Quantitative PCRs were performed in a ABI Prism 7000 Sequence Detection System (Applied Biosystems). Reactions contained 0.3 μM of each primer, 1 × Power SYBR Geen Master Mix (Applied Biosystems), 4 μl cDNA sample and distilled water in a total reaction volume of 30 μl. Thermal cycles were: 50°C; 2 min, 95°C; 10 min, 40 cycles: 95°C; 15 s, 60°C; 1 min. To ensure that single PCR products of intended lengths were amplified, a melting program was executed subsequent to the quantifications and PCR products were separated by gel electrophoresis. To control for potential signals from amplification of genomic DNA (gDNA) during quantification, minus RT controls were employed (reverse transcription without M-MuLV RNase H- reverse transcriptase). gDNA signals were consistently undetectable or negligible. *Data analysis: *Background-subtracted expression values (copy numbers) were determined relative to a standard curve (cDNA dilution series) in 7000 SDS v.1.2.3 (Applied Biosystems). Expression values in each sample were normalized to the geometric mean of the endogenous expression of *Actb *and *Gapdh*. The criterion for differential expression was p ≤ 0.05, two-tailed unequal variance t-test (R v2.6.2 Environment for Statistical Computing http://www.r-project.org, t.test(), Arguments: alternative = "two.sided", var.equal = FALSE).

### Sequential RNA - DNA FISH

*Cells:* Adult mouse fibroblast cultures were prepared as previously described [69] with minor modifications. Briefly, female mice were decapitated and the tails were cut and washed in PBS. After removing superficial dermis, remaining tails were cut into 2-3 mm pieces and placed into gelatin-coated 6 well plates (Costar) containing 1 ml medium in each well. Culture medium was composed of Dulbecco's Modified Eagle Medium (DMEM) containing 4.5 g/l glucose, 10% fetal bovine serum and 0.5% penicillin/streptomycin (Gibco). Tails were incubated at 37°C and 5% CO_2 _for 5 days, during which fibroblasts migrated out of the explants. Tissue pieces were then removed and cells were cultured in fresh medium until they reached confluence. *RNA - DNA FISH: *Mouse fibroblasts were passaged and grown on Culturewell™ MultiWell cell culture system (Molecular Probes) for 36 hours, and then fixed by 3% paraformaldehyde for 15 min at room temperature, followed by permeabilization in 0.5% TritonX-100 in PBS with 10 mM Ribonucleoside Vanadyl Complex (New England Biolabs) for 5 min. Fixed cells were stored in 70% Ethanol at -20°C until usage. Sonicated BAC DNA (*5530601H04Rik *and *2610029G23Rik*: RP23-149J5, *Xist: *RP23-84A16) was labelled with Green-dUTP or Orange-dUTP (Abbott Molecular) using the BioPrime Array CGH Genomic Labeling system (Invitrogen). Hybridization with labeled DNA (10 ng/μl) and mouse Cot1 DNA (100 ng/μl) (Invitrogen) was performed overnight at 37°C in 2 × SSC, 50% formamide and 12% dextran sulfate, with 10 mM Ribonucleoside Vanadyl Complex. Cells were washed with 2 × SSC and 50% formamide (15 min; 40°C) and 2 × SSC (15 min; 40°C). DNA was counterstained and cells were mounted in Vectashield (Vector Labs). Cell imaging and generation of optical sections in 3D were carried out on Leica DMI 3000B. After image acquisition, the slide with cells were washed two times by 4 × SSC with 0.05% Tween20 (10 min; 40°C), followed by treatment with 10 ng/μl RNase A (QIAGEN) at 37°C for 1 h. Cells were then denatured in 2 × SSC and 50% formamide at 80°C for 40 min. DNA FISH hybridizations were carried out with labelled DNA (10 ng/μl) and mouse Cot1 DNA (100 ng/μl) (Invitrogen) in 2 × SSC, 50% formamide and 12% dextran sulfate overnight at 37°C, followed by the same washing and mounting steps as described for RNA FISH. DNA FISH images, of cells with recorded RNA FISH signals, were acquired in the same way. The images presented in Figure 5 were uniformly processed and merged in Adobe Photoshop CS3 (Adobe).

### H3K27me3 ChIP-chip data

H3K27me3 data from female and male adult mouse liver [43] was downloaded from NCBI Gene Expression Omnibus (GEO accession: GSE20617, data sets: GSM517918_female_liver _H3K27me3_peaks, GSM517917_male_liver_H3K27me3_peaks). LiftOver (UCSC Genome Browser) and SignalMap v1.9.0.03 (NimbleGen) was used to visualize H3K27me3 enrichment shown in Figure 6.

## Competing interests Statement

The authors declare that they have no competing interests.

## Authors' contributions

BR and EJ conceived and coordinated the study. BR, EJ and KSS wrote the manuscript with assistance from all authors. GDR, LL and RWW prepared the microarray data. BR analysed the microarray data. BR and KK prepared tissues for the qPCR experiments. BR and LH performed the qPCR experiments. BR and KJR established the fibroblast culture. BR and CS performed the RNA-DNA FISH experiments. BR and KSS analysed the ChIP-chip data, and jointly hypothesized mechanisms involving lncRNAs in X-inactivation escapee domains. All authors read and approved the final manuscript.

## Supplementary Material

Additional file 1 **Significant probes**. Click here for file

Additional file 2 **IPA**. Click here for file

Additional file 3 ***Xist *scatter plots**. Click here for file

Additional file 4 **Primers**. Click here for file
