# Supplementary material for: Female-biased expression of long non-coding RNAs in domains that escape X-inactivation in mouse
Source: BMC Genomics. 2010 Nov 3;11:614. doi: 10.1186/1471-2164-11-614 (PMC3091755; doi:10.1186/1471-2164-11-614)
Supplement: Additional file 3 — Xist scatter plots. [file 1471-2164-11-614-S3.PDF]

Additional file 3.

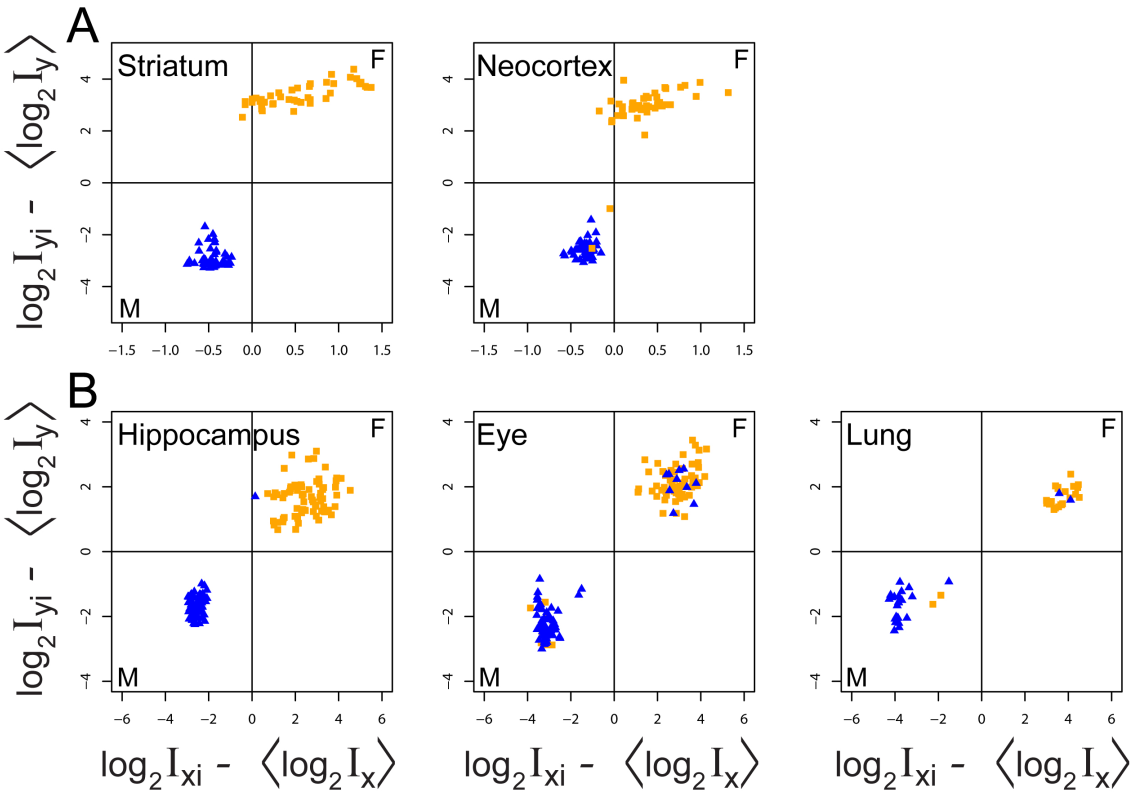

Exclusion of arrays prior to sex-specific analysis

Prior to any further analysis, we screened the data collection for arrays in which sex was possibly mislabelled, since such misclassifications would reduce power in the sex-specific analysis. This was done by plotting the $\log_2$ intensity (I) values of two *Xis*t probes in each array (i) as relative to the overall mean intensity ($<\log_2 I>$) of the probe in the tissue. Female labelled (orange squares) and male labelled (blue triangles) arrays in which *Xist* expression fell in the quadrant of the opposite sex in the plot were classified as potentially mislabelled and were excluded from the subsequent sex-specific analysis. The *Xist* probes were **A:** Illumina Mouse-6v1.1, x; scl0213742.1_91-S, y; scl00213742.1_141-S, **B:** Affymetrix M430v2, x; 1427262_at, y; 1436936_s_at.
